# Increased medial olivocochlear reflex strength in normal-hearing, noise-exposed humans

**DOI:** 10.1371/journal.pone.0184036

**Published:** 2017-09-08

**Authors:** Ishan Bhatt

**Affiliations:** Department of Communication Sciences & Disorders, Northern Arizona University, Flagstaff, AZ, United States of America; University of South Florida, UNITED STATES

## Abstract

Research suggests that college-aged adults are vulnerable to tinnitus and hearing loss due to exposure to traumatic levels of noise on a regular basis. Recent human studies have associated exposure to high noise exposure background (NEB, i.e., routine noise exposure) with the reduced cochlear output and impaired speech processing ability in subjects with clinically normal hearing sensitivity. While the relationship between NEB and the functions of the auditory afferent neurons are studied in the literature, little is known about the effects of NEB on functioning of the auditory efferent system. The objective of the present study was to investigate the relationship between medial olivocochlear reflex (MOCR) strength and NEB in subjects with clinically normal hearing sensitivity. It was hypothesized that subjects with high NEB would exhibit reduced afferent input to the MOCR circuit which would subsequently lead to reduced strength of the MOCR. In normal-hearing listeners, the study examined (1) the association between NEB and baseline click-evoked otoacoustic emissions (CEOAEs) and (2) the association between NEB and MOCR strength. The MOCR was measured using CEOAEs evoked by 60 dB pSPL linear clicks in a contralateral acoustic stimulation (CAS)-off and CAS-on (a broadband noise at 60 dB SPL) condition. Participants with at least 6 dB signal-to-noise ratio (SNR) in the CAS-off and CAS-on conditions were included for analysis. A normalized CEOAE inhibition index was calculated to express MOCR strength in a percentage value. NEB was estimated using a validated questionnaire. The results showed that NEB was not associated with the baseline CEOAE amplitude (r = -0.112, *p* = 0.586). Contrary to the hypothesis, MOCR strength was positively correlated with NEB (r = 0.557, *p* = 0.003). NEB remained a significant predictor of MOCR strength (*β* = 2.98, *t*(19) = 3.474, *p* = 0.003) after the unstandardized coefficient was adjusted to control for effects of smoking, sound level tolerance (SLT) and tinnitus. These data provide evidence that MOCR strength is associated with NEB. The functional significance of increased MOCR strength is discussed.

## Introduction

Noise-induced hearing loss (NIHL) remains a hearing health concern despite Occupational Safety and Health Administration standards for hearing protection and public health awareness campaigns. NIHL affects approximately 15% of Americans aged 20 to 60 years; and it is a frequently occurring disability among current combat veterans [1]. Several studies have found that youth are exposed to potentially hazardous levels of recreation noise which may lead to NIHL [2–5]. Recent reports suggest that NIHL is no longer limited to industrial workers exposed to loud noise but is found in children, adolescents and college-aged young adults [6–8].

The college-aged adults are routinely exposed to damaging sound levels [4, 5]. Almost 90% of adolescents report that they listen to music on a regular basis, with 26% listening to music for more than 3 hours per day, and 48% of them reporting that their typical listening level is at a high or near-to-maximum volume [4, 5]. Personal music players have been shown to exceed damaging sound pressure levels at high volume control settings [9]. Research on the auditory lifestyle of college students has shown that almost 50% were exposed to potentially harmful music, 44% used noisy equipment without hearing protection, and almost 29% of them worked in a noisy environment suggesting that the population might be susceptible to hearing loss and tinnitus [10].

Cochlear hair cells are widely accepted as the most vulnerable cochlear structure to noise-induced damage. Noise-induced hair cell damage can cause reduction in hearing sensitivity [11, 12] which can produce a notch at 3 to 6 kHz in a behavioral audiogram [13–15]. Behavioral audiometry is commonly considered a “gold standard” test for identifying NIHL. This view has been challenged by recent research which demonstrated that noise exposure can lead to auditory neural degeneration, in some cases a loss of up to 50% of the synapses between inner hair cells and auditory afferent neurons can be detected, even when physiology of the hair cells recovers and hearing thresholds and otoacoustic emissions (OAEs) return to normal [16–18]. The cochlear synaptopathy is referred to as “hidden hearing loss” because this neural damage cannot be detected by behavioral hearing thresholds or by OAEs and electrophysiological thresholds until it becomes severe [19–20].

Recent human studies found evidence of cochlear synaptopathy in noise-exposed normal hearing humans [20–22]. Auditory functions were investigated in young adults with hearing thresholds ≤ 15 dB HL at the conventional audiometric frequencies and who were exposed to a range of noise exposure background (NEB) [21]. NEB was defined as the amount of noise exposure an individual has encountered in daily life. NEB was estimated using a validated questionnaire which inquired about the frequency (‘how often’) and duration (‘how long’) of noise exposure in ‘routine’ (e.g. home, travel etc.) and ‘episodic’ activities (e.g. power tools, attending sporting events etc.). The study obtained a significant negative correlation coefficient between NEB and wave I amplitude of auditory brainstem responses (ABR). Individuals with high NEB showed lower wave I amplitude than individuals with low NEB. The relationship was stronger for ABR elicited by high intensity stimuli, and was weakened and disappeared as the stimulus intensity was reduced. A similar study found that individuals with high NEB showed elevated behavioral hearing thresholds at ultra-high frequencies, elevated summating potential and action potential ratio, poor performance on word recognition in noise and heightened reaction to sounds [20]. These studies found no relationship between NEB and behavioral hearing thresholds or OAE amplitudes at the conventional audiometric frequencies (250–8000 Hz).

While the effects of NEB on the auditory afferent system are studied in the literature, the effects of NEB on the auditory efferent system largely remains unknown. The medial olivocochlear (MOC) system is one of two efferent systems that influence cochlear functioning [23]. In the medial olivocochlear reflex (MOCR) circuit, auditory nerve fibers connect to a subpopulation of multipolar neurons in the posteroventral cochlear nucleus which further connect to the medial part of the superior olivary complex in the auditory brainstem and projects bilaterally to the outer hair cells (OHCs) of the cochleae [24–30]. The MOCR circuit regulates cochlear gain by modifying activity of OHCs and subsequently modulating activity of the auditory nerve [30, 31]. The MOCR circuit has been associated with frequency discrimination, selective attention, speech perception in noisy situations, protecting the cochlea from noise and aging; and with tinnitus and sound level tolerance (SLT) [32–39].

The aim of the present study was to investigate the relationship between NEB and the strength of the MOCR in a sample of college-aged adults with normal hearing sensitivity and with a range of NEB. It was hypothesized that subjects with high NEB would exhibit reduced afferent neural input [20–22] to the MOCR system which would subsequently lead to reduced strength of the MOCR. In turn, it will result in a negative correlation between the strength of the MOCR and NEB. Numerous studies have investigated the MOCR by using Click-evoked OAEs (CEOAEs) in humans (e.g. [40, 41]). The present study controlled several methodological factors important to measure the MOCR using CEOAEs [23, 30, 32], e.g. (1) recording CEOAEs with linear clicks, (2) control for influence of middle ear muscle reflex (MEMRs) on CEOAEs, and (3) measurement of the normalized CEOAE inhibition index.

## Materials and methods

### Ethics statement

The Institutional Review Board of Northern Arizona University has reviewed and approved the study protocol. Subjects were recruited from students enrolled at the Flagstaff Mountain Campus of the Northern Arizona University. A written and informed consent was obtained for each subject prior to the data collection process.

### Subjects

A recruitment flyer was distributed in a class of 54 undergraduate students at the Flagstaff campus of Northern Arizona University. The students were instructed to contact the investigator to participate in the study. A sample of 40 human adults (14 males and 26 females) aged 18 to 35 years was recruited. An otoscopic exam was performed on all participants. Those with normal otoscopic findings were tested with pure tone audiometry. All audiometric measures described in this study were collected in a sound treated booth meeting ANSI standards (ANSI S3.1–1999). Audiometric thresholds were obtained at 250, 500, 1000, 2000, 3000, 4000, 6000 and 8000 Hz (GSI-61, Eden Prairie, MN) with ER-3A insert receivers (Etymotic Research. Inc, Elk Grove Village, IL), using the modified Hughson-Westlake procedure. Participants with hearing thresholds ≤ 15 dB HL at each audiometric frequency were tested with tympanometry. Tympanometry was performed using a 226 Hz probe tone presented through Titan IMP440 (Interacoustics, Middelfart, Denmark). Participants with normal tympanograms (static compliance between 0.35 to 1.75 cc and peak pressure value between +50 to -100 daPa) in both ears were considered for further testing. Along with otoscopy and tympanometry, an informal interview was conducted to rule out active middle ear pathologies. Subjects reporting systemic diseases, neurological or immunological disorders were excluded from the study. Eleven subjects who did not meet the above listed criteria were excluded from the further testing.

### Middle ear muscle reflex (MEMR)

**Fig 1:**
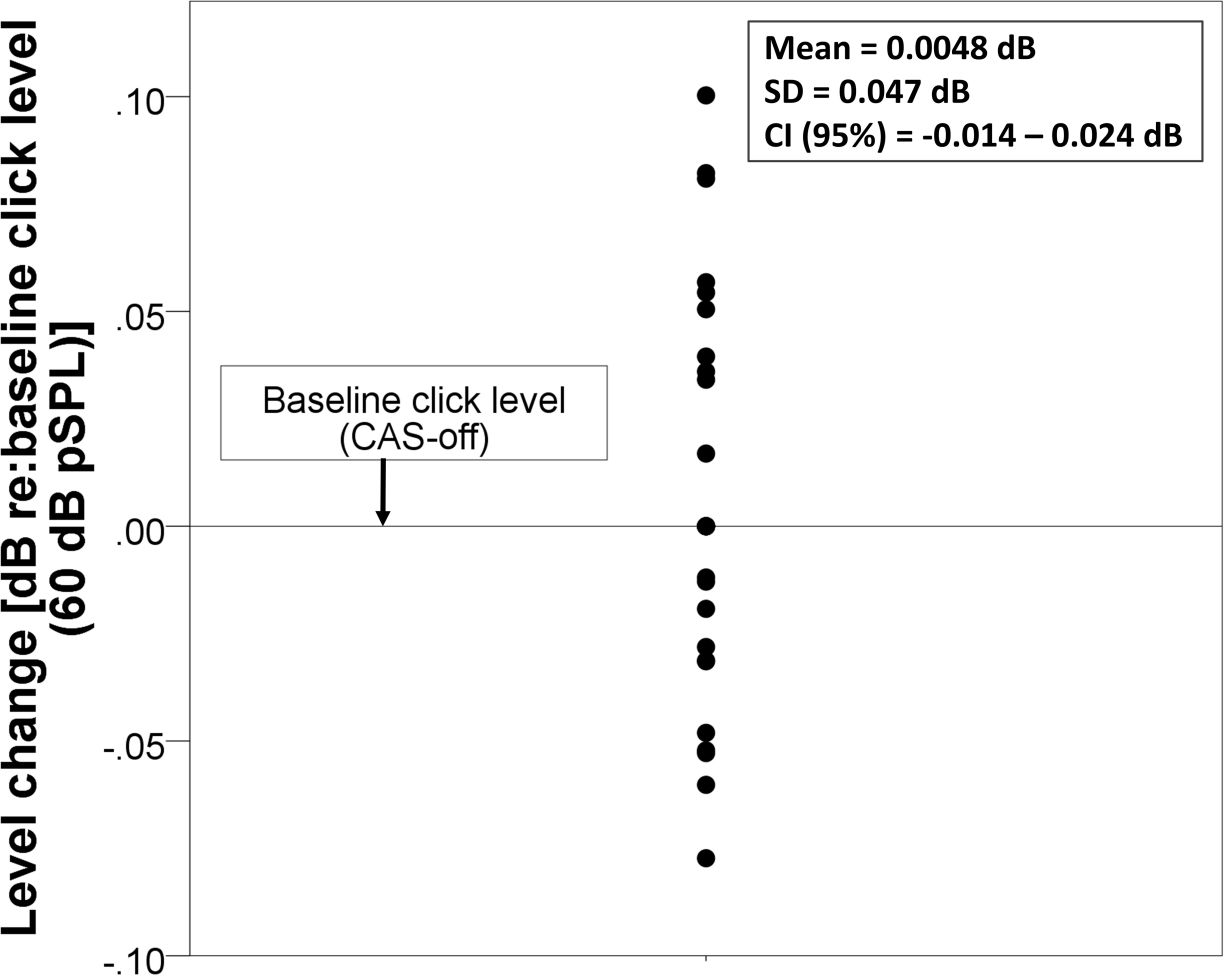
Results of MEMR test. Mean and the corresponding individuals data for the change in stimulus level with reference to baseline CAS-off condition (dB) is plotted (Y-axis). Black straight line at 0 dB presents normalized baseline stimulus level (in CAS-off condition). Black symbols are individuals means of RMS amplitude near the stimulus trough.

MEMR testing was performed to determine whether the level of contralateral acoustic stimulation (CAS) used to measure the MOCR (e.g. 60 dB SPL) could evoke MEMRs. The initial presentation level was 60 dB SPL and the stimulus intensity was increased in 5 dB steps. MEMR was defined as the lowest BBN level in the contralateral ear (i.e. right ear) at which a reduction of at least 0.02 cc in middle ear compliance could be measured on two trials. All subjects included in the study had a contralateral MEMR threshold ≥ 80 dB SPL for BBN elicitor. The mean MEMR threshold was 95 dB SPL [standard deviation (SD) = 6 dB SPL]. The CAS used to measure the MOCR was lower than the MEMR thresholds by a mean of 35 dB. However, it is known that the actual MEMR thresholds could be almost 12 dB lower, as measured by the wideband acoustic reflectance method [42, 43]. Therefore, an additional method to control for the effect of low level MEMRs on the MOCR was applied. This approach is based on the assumption that a low level MEMR would increase the level of the stimulus (i.e. click) in the ear canal by stiffening the ossicular chain [44–47]. To test for the presence of low level MEMR, 60 dB pSPL clicks presented at 19.3 Hz were used as probe stimuli. Clicks were recorded in the ear canal in two conditions, one without any contralateral elicitor and one with the same contralateral elicitor used in the study to elicit MOCR. RMS levels of the ear-canal recorded clicks were obtained for every participant in a time window (100 μs long) near first trough of the click waveform for CAS on/off conditions. The first trough is the largest deviation in sound pressure measured in the ear canal that occurs due to ringing of the click stimulus [45]. The observed changes were expected to be smaller compared to level changes that would be expected if the MEMR was activated, i.e. >1.4% (0.12 dB) [44–47]. As seen in Fig 1, participants showed an average 0.0048 ± 0.047 dB deviation in the presence of the MOC elicitor (re: baseline CAS-off condition). Participants with > 1.4% of increase in the stimulus level were excluded to assure no or minimum effect of low level MEMR on the CEOAE inhibition measurement. This criterion led to the exclusion of one subject from the statistical analysis.

### Initial cochlear screening through CEOAEs

CEOAEs were measured using the SmartTrOAE (version 5.10, Intelligent Hearing System, Florida, USA) connected with ER-10D probe (Etymotic Research. Inc, Elk Grove Village, IL). CEOAEs were elicited in a non-linear mode with 75 μs clicks presented at 80 dB pSPL (±0.3 dB, calibration in an IEC-711 ear simulator using the peak-equivalent method with a 1 kHz reference tone) at the rate of 19.3/s. Recordings were time windowed from 2 to 20 ms with the sampling period of 25 μs. Responses to a total of 1024 sweeps were averaged. The noise rejection level of 47 dB was utilized to record the CEOAEs. The SmartTrOAE averages into two alternative buffers: A and B. Signal is estimated from the (A+B)/2 waveform and noise is estimated from the A-B difference waveform. The in-ear probe calibration test as recommended by SmartTrOAE software was performed. This software calibrates the stimulus level in the ear canal at the beginning of testing and subsequently monitors the level throughout the response acquisition period to display stimulus stability. The stimulus stability displayed by the instrument was used by the tester to monitor adequacy of probe placement and subsequently the stimulus level across the entire testing procedure. The SmartTrOAE software estimates reproducibility of CEOAE responses by calculating the cross-correlation between buffer A and B. CEOAE responses were accepted only if the overall reproducibility and stimulus stability exceeded 90%.

### CEOAE-based assay of MOCR inhibition

The MOCR was measured on one ear per subject. The left ear was chosen because it has been associated with a higher susceptibility to NIHL [48]. Two sets of CEOAEs were elicited in a linear mode with 75 μs clicks presented at 60 dB pSPL (±0.3 dB; calibration in an IEC-711 ear simulator using the peak-equivalent method with a 1 kHz reference tone) at the rate of 19.3/s: one with CAS-off and another with CAS-on. In the CAS-on condition, continuous BBN (0.125–8 kHz) was presented through the SmartTrOAE software connected with electrically shielded Etymotic ER-3A insert earphones (Etymotic Research. Inc, Elk Grove Village, IL). Recordings were time windowed from 2 to 20 ms with the sampling period of 25 μs. Responses to a total of 1024 sweeps were averaged. The noise rejection level of 47 dB was used to measure the CEOAEs. The in-ear probe calibration test was performed as recommended by SmartTrOAE software. The stimulus stability displayed by the instrument was used by the tester to monitor adequacy of probe placement and subsequently the stimulus level across the entire testing procedure. CEOAE responses were accepted only if the overall reproducibility and stimulus stability exceeded 90%. These two sets of recordings were repeated with a probe tip inserted in the same position. The participants were instructed to stay awake and as still as possible during the testing session. They were instructed to keep their head and eye gaze straight during the entire CEOAE measurement. Participants were not instructed to attend or ignore the test stimuli. Participants with a SNR value of at least 6 dB in the overall CEOAE response with CAS-off and CAS-on conditions were included in the statistical analysis. This criterion led to the exclusion of two subjects from statistical analysis.

### Quantification of MOCR strength

The MOCR strength was quantified using a normalized index (ΔCEOAEn). The normalized index (ΔCEOAEn) was calculated by converting CEOAE amplitude into a linear micropascal scale. The CEOAE amplitude in the CAS-on condition was subtracted from the CEOAE amplitude in the CAS-off condition to calculate ΔCEOAE. The normalized index (ΔCEOAEn) was quantified as percentage change from the baseline amplitude. The normalized index was computed according to the formula, $\Delta CEOAEn=\frac{\Delta CEOAE}{CEOAEbaseline}\times 100$. It was argued that referencing to baseline amplitude eliminates biases related to inter-subject differences in magnitude of the CEOAE [32, 49]. Positive values denote MOC inhibitory effects on CEOAE. The higher percentage values indicate greater MOCR strength and lower values indicate lower MOCR strength.

### Questionnaire

A questionnaire was constructed to estimate audiological factors and NEB (S1 Appendix). This survey included assessment of five major areas: demographic details, routine acoustic exposure, SLT, tinnitus and smoking. (1) Demographic details: Participants were asked about their age, gender and ethnicity. (2) Routine acoustic exposure: Acoustic exposure was estimated via a self-report questionnaire [50]. This survey has been validated to estimate overall acoustic exposure and has been utilized in previous research to quantify noise exposure in young adults [21, 22, 50]. It assessed nine specific known areas of high acoustic exposure. These included exposure to six areas of noise exposure: occupational noise, power tools, heavy equipment, commercial sporting or entertainment events, motorized vehicles, small aircraft; and three areas of music exposure: music instrument playing, music listening via personal earphones, and music listening via audio speakers. The survey included questions about frequency (i.e. how often) and duration (i.e. how long) of noise exposures. The responses were elicited using a forced choice method. Responses were rated categorically to calculate the overall noise dose which was reported as L_Aeq8760h_. Here, “L” represents sound pressure level measured in dB, “A” presents use of an A-weighted frequency response, “eq” represents a 3-dB exchange rate for calculation of the time/level relationship, and “8760h” represents the total duration of noise exposure in hours over one year (365 days/year X 24 hours/day). Further details of the survey can be found elsewhere [21, 50]. (3) Sound level tolerance (SLT): Assessment was conducted by three questions as follows: “Many everyday sounds are unbearably loud to me”; “Sounds that others believe are moderately loud are too loud for me”; and “I hear very soft sounds that others with normal hearing do not hear”. Participants were asked to rate agreement with these sentences on a 0 to 100 scale, where 0 = completely disagree and 100 = completely agree. Ratings of these questions were averaged to quantify SLT on a 0 to 100 scale. These questions were used for quantifying SLT in previous studies [37, 51–53]. (4) Tinnitus: The questions inquiring about tinnitus were adopted from the National Health and Nutrition Examination Survey [54]. This section inquired about tinnitus with an opening question: “In the past 12 months, have you been bothered by ringing, roaring, or buzzing in your ears or head that lasts for 5 minutes or more?”. If the participant answered positively to this question, then the follow-up question was: “How long have you been bothered by this ringing, roaring, or buzzing in your ears or head?”. Response choices for this question included: < 3 months/3 months to a year/1-4 years/5-9 years/≥ 10 years/Don’t know. If the participant answered negatively to the first question, then the follow-up question was: “Have you ever experienced ringing, roaring, or buzzing in your ears or head?”. Response choices for this question included: Yes, No, Don’t know. Tinnitus was classified into two categories: No tinnitus experience in a lifetime and at least one tinnitus experience in a lifetime. (5) Smoking: This section inquired about smoking with an opening question: “Do you or have you smoked tobacco?” If the participant answered positively to this question, then the follow-up question was: “What types of smoking do you prefer, or have preferred, on a regular basis? (percentage values of all selected choices must add up to 100%)”. Smoking was classified into two categories: present or absent smoking history.

### Statistical analysis

Data were analyzed in the SPSS software (version 23.0; SPSS, INC). Statistical analysis was performed on 26 participants following all of the inclusion criteria described above. A linear regression model was utilized to quantify the relationship between overall ΔCEOAEn(%) and NEB. Two models were constructed to achieve this aim: (1) model 1: overall ΔCEOAEn(%) was included as a continuous dependent variable and NEB (in LAeq8760hrs) as a continuous independent variable; (2) model 2: overall ΔCEOAEn(%) was included as a continuous dependent variable and noise (in LAeq8760hrs), gender, ethnicity, smoking, SLT and tinnitus as independent variables. Model 2 allowed controlling for variables such as smoking, SLT and tinnitus which has documented effects on CEOAE inhibition and NIHL [6, 8, 37, 55–58].

## Results

### Demographic and audiological details of the sample

Table 1 provides demographic and audiological details of the study sample. A total of 26 subjects, 8 males and 18 females, met the inclusion criteria (detailed in methods). Twenty-one participants reported non-Hispanic European American racial ancestry. Thirteen subjects reported that they had experienced tinnitus. Seven participants reported that they smoked tobacco and five participants reported a low SLT score (score < 50). None of the participants reported hearing loss.

**Table 1:** Demographic and audiological details of the study sample (N = 26).

Variables		Frequency	
Gender			
Male		8 (30.8%)	
Female		18 (69.2%)	
Ethnicity/predominant racial ancestry			
	Non-Hispanic European American	21 (80.7%)	
	Others or multiracial	5 (19.3%)	
Have you ever experienced ringing, roaring, or buzzing in your ears or head?			
	Yes	13 (50%)	
	No	13 (50%)	
Do you or have you smoked tobacco?			
	Yes	7 (26.9%)	
	No	19 (73.1%)	
Sound Level Tolerance (SLT)			
	High (score ≤ 50)	21 (80.7%)	
	Low (score > 50)	5 (19.3%)	
Self-reported hearing loss			
	Yes	0 (0%)	
	No	26 (100%)	

### Correlation between NEB and the baseline physiologic measures

**Fig 2:**
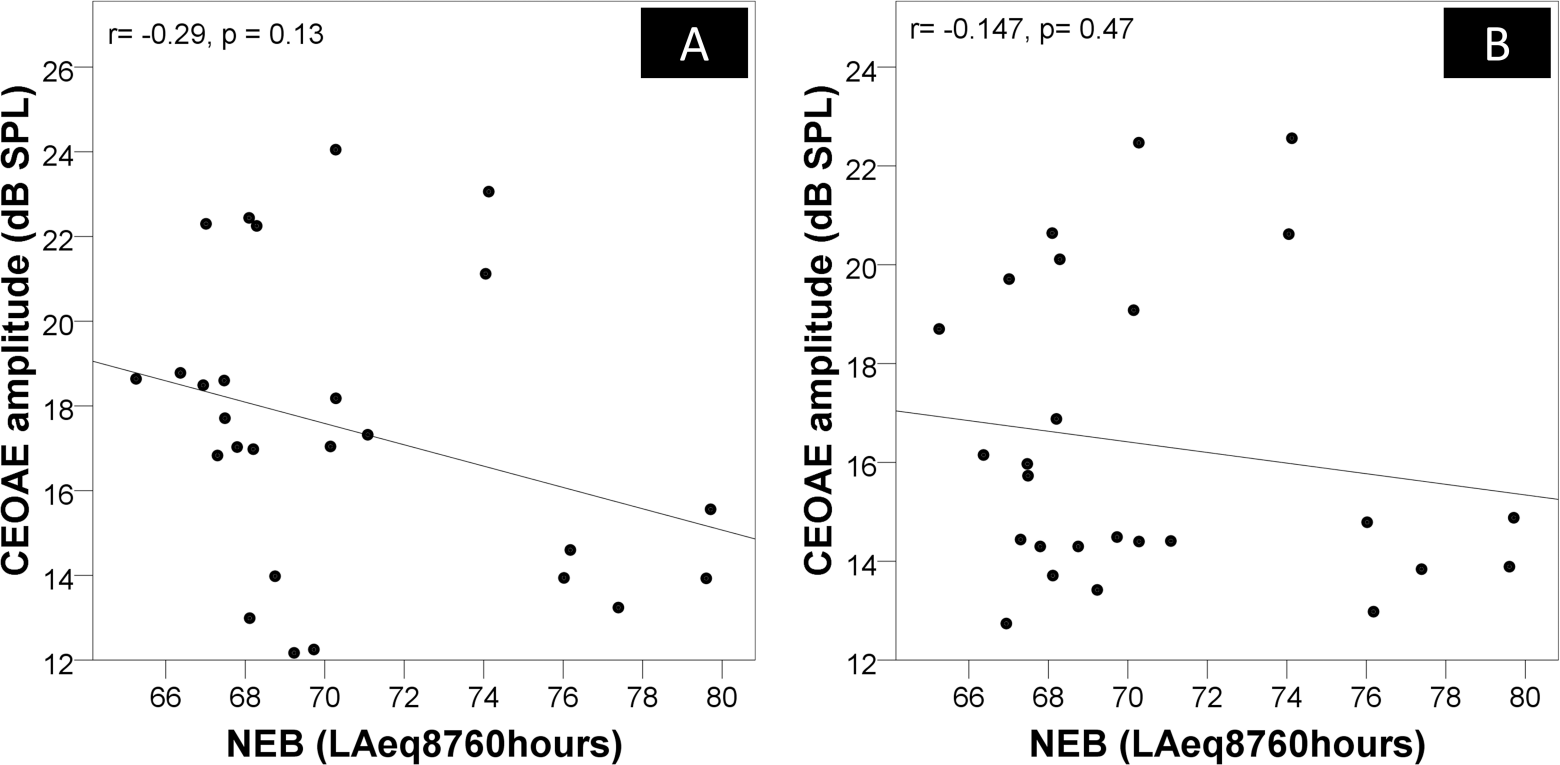
Relationship between NEB and CEOAE amplitude. (A) Scatter plot between NEB and the baseline CEOAE amplitude elicited by 80 dB pSPL non-linear clicks. A linear regression line is inserted to show the predictive relationship. Pearson’s correlation coefficient (r) and *p* value are inserted on the top left corner of the plot. (B) Scatter plot between NEB and the CEOAE amplitude elicited by 60 dB pSPL linear clicks in CAS-off condition. A linear regression line is inserted to show the predictive relationship. Pearson’s correlation coefficient (r) and *p* value are inserted on the top left corner of the plot.

The data revealed no significant correlation (*p*<0.05) between NEB and hearing thresholds at any audiometric frequency in both ears (Table 2). There was no significant correlation between NEB and the contralateral acoustic reflex threshold elicited by BBN (r = 0.238, *p* = 0.24). No significant correlation was found between NEB and overall baseline CEOAE amplitude measured by non-linear clicks (r = -0.112, *p* = 0.586) (Fig 2).

**Table 2:** Pearson product-moment correlation coefficients for NEB and hearing thresholds.

	Correlation coefficients								
	Hearing thresholds (Hz)	250	500	1000	2000	3000	4000	6000	8000
NEB	Left ear	0.20	0.19	0.36	0.11	0.18	0.15	0.10	-0.02
	Right ear	0.18	-0.02	0.09	-0.04	-0.003	0.02	0.11	0.11

None of the coefficients were statistically significant (*p* ≤ 0.05)

### ΔCEOAEn(%) index

The average SNRs of CEOAE recordings in CAS-off and CAS-on were 9.3 dB (SD = 2.55) and 8.8 dB (SD = 2.25), respectively. The mean raw dB effect and normalized index for ΔCEOAEn(%) were 0.78 dB (SD = 1.45) and 7.37% (SD = 15.07), respectively. The MOC inhibition data followed a normal distribution as determined by the Shapiro-Wilk test (*W* statistics = 0.969, *p* = 0.59).

### Relationship between NEB and ΔCEOAEn(%)

**Fig 3:**
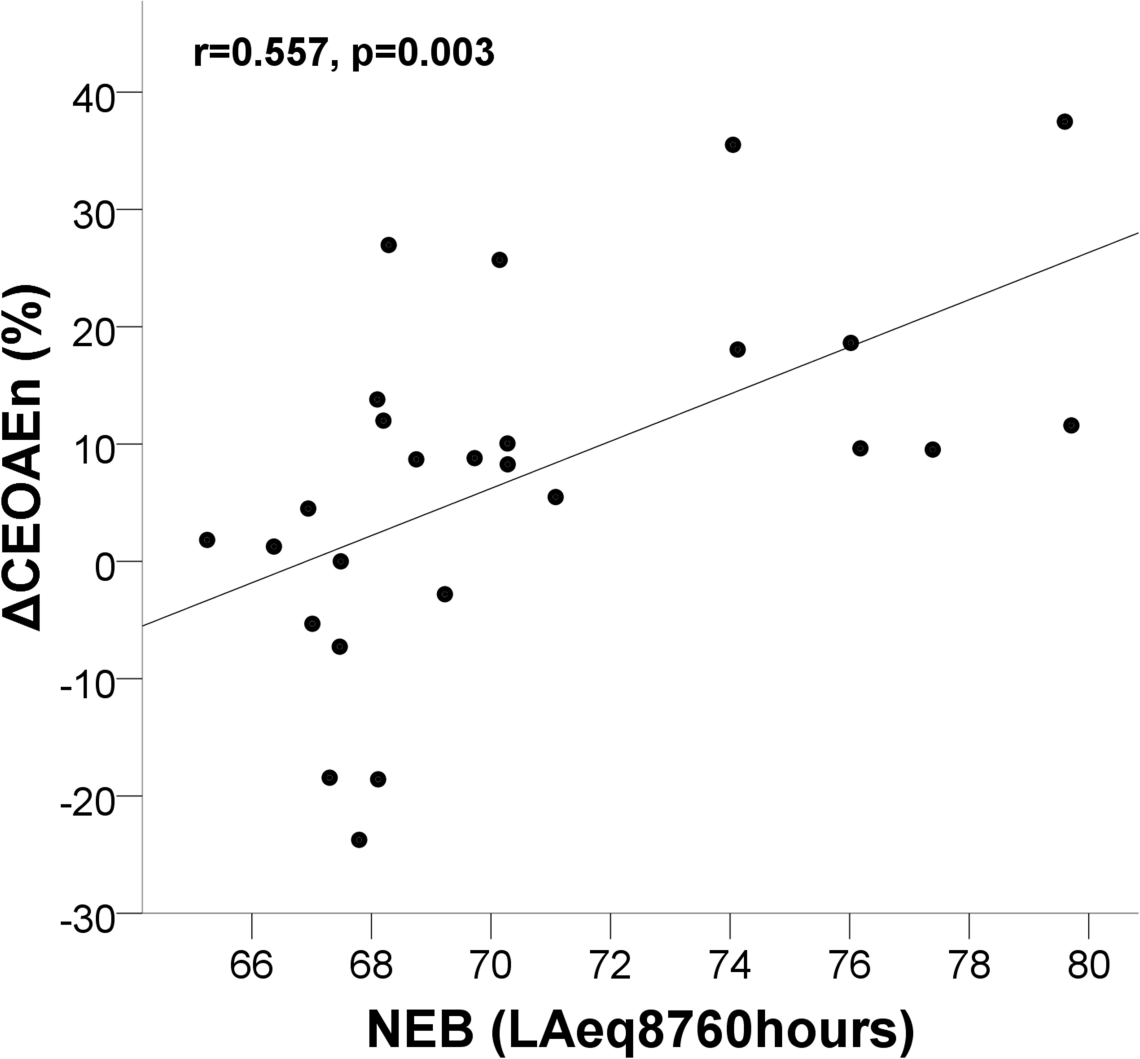
Relationship between NEB and MOC inhibition. Scatter plot between NEB and overall ΔCEOAEn(%). A linear regression line is inserted to show the predictive relationship. Pearson’s correlation coefficient (r) and *p* value are inserted on the top left corner of the plot.

The correlation coefficient for NEB and ΔCEOAEn(%) was statistically significant (r = 0.557, *p* = 0.003) (Fig 3). Model 1 revealed a significant relationship between NEB and overall ΔCEOAEn(%) with *β* = 2.011, *t*(24) = 3.287, *p* = 0.003. NEB explained a significant proportion of variance in overall ΔCEOAEn(%), adjusted *R*^*2*^ = 0.282, *F*(1,24) = 10.80, *p* = 0.003. Model 2 revealed that smokers have significantly lower ΔCEOAEn(%) than non-smokers (*β* = 21.17, *t*(19) = 2.338, *p* = 0.03). NEB showed a significant relationship with overall ΔCEOAEn(%) (*β* = 2.98, *t*(19) = 3.474, *p* = 0.003) when the unstandardized coefficient was adjusted to control for the effects of smoking, SLT, tinnitus, gender and ethnicity on overall ΔCEOAEn(%). Tinnitus (*β* = 5.499, *t*(19) = 0.951, *p* = 0.35), SLT (*β* = -0.15, *t*(19) = -0.126, *p* = 0.90), gender (*β* = -8.0, *t*(19) = -1.115, *p* = 0.27) and ethnicity (*β* = -6.727, *t*(19) = -0.966, *p* = 0.34) did not show association with the overall ΔCEOAEn(%). Model 2 explained a significant proportion of variance in overall ΔCEOAEn(%) with adjusted *R*^*2*^ = 0.334, *F*(1,19) = 3.08, *p* = 0.028. Fig 3 describes the relationship between NEB and ΔCEOAEn(%).

## Discussion

The major finding of this study is that NEB is positively correlated with the strength of the MOCR. The study reports MOC inhibition data after considering important MOC measurement issues discussed in the previous work [23, 30, 32, 47, 49]. This study utilized a validated questionnaire to estimate routine noise exposure in young adults [50]. The NEB estimated using this questionnaire has been associated with reduced amplitude of ABR wave I in individuals with clinically normal hearing and a high NEB score [21]. Contrary to the hypothesis, the results of the study showed that individuals with high NEB exhibited increased strength of the MOCR. In addition, the study found that the strength of the MOCR was reduced in smokers compared to non-smokers. This finding is in agreement with previously published reports which further validate the methods used in the present work [55–58]. Identification of increased MOCR strength in normal hearing subjects with high NEB provides an opportunity to discuss the influence of NEB on the auditory efferent system.

The influence of NEB on the strength of the MOCR has not been investigated well in the literature. A recent study evaluated the MOCR strength in musicians with a range of years spent in formal musical training [59]. The study defined musicians as instrumentalists who had received ≥ 5 years of continuous private instruction on their principal instrument, beginning prior to age 12, and were currently active in music training or practice. Non-musicians were defined as individuals with ≤ 3 years of self-directed music training with no instruction within the past 5 years. The study found that musicians exhibited increased strength of the MOCR compared to non-musicians, and a positive correlation was obtained between MOCR strength and years spent in formal musical training. The authors concluded that the MOCR can be strengthened by musical training. The present study may offer an alternative explanation for the observed association. Numerous studies have reported that musicians are exposed to loud traumatic sounds on a regular basis [60–62]. It is conceivable that musicians are likely to exhibit higher NEB than non-musicians which may subsequently influence the MOCR strength. According to the criteria described by the authors [59], all participants in the present study were classified as non-musicians. The present study showed that increased MOCR strength can be found in non-musicians with high NEB.

Musical training has been shown to have a profound impact on auditory skills, improving not only basic perceptual acuity for speech sound but also the brain’s ability to extract important communication signals from background noise [63–69]. It has been suggested that increased MOCR strength in musicians may contribute to their ability to improve speech perception in noise [59]. A number of studies have been conducted to investigate the relationship between MOCR strength and speech perception ability; however, the relationship still remains largely elusive [70–76]. Some studies found a significant relationship [70–72]; while others found no relationship between strength of MOCR and speech perception in noise [73–76]. A study controlling important methodological factors found that MOCR strength was not related to speech perception in noise; it was concluded that the auditory system may not employ the MOC system in a reflexive manner to improve speech perception in noise [32]. It is conceivable that musicians use the MOC system in a non-reflexive manner to exert fine control over cochlear functioning to improve speech perception. Therefore, it is likely that the increased MOCR strength in musicians might not influence their ability to extract important communication signals from background.

### Possible association between NEB, tinnitus, SLT and increased MOCR strength

Tinnitus and SLT are frequently co-occurring auditory disorders which might share some common underlying neural substrates. The role of MOCR strength in patients with tinnitus and SLT was investigated in a precious research[37]. The study revealed increased MOCR strength in patients suffering from tinnitus and/or SLT. It was concluded that the increased MOCR might be associated with a common underlying process driving MOC inhibition in tinnitus and SLT. In the present study, NEB remained a significant predictor of MOCR strength after controlling for the effect of tinnitus and SLT. Therefore, it is plausible that the increased MOCR strength found in patients with tinnitus and/or SLT might be secondary to their exposure to high NEB which is a widely accepted risk factor for tinnitus and SLT.

The potential mechanisms underlying the increased MOCR strength has been speculated in the previous research [37] which included (1) increased responsiveness of MOC interneurons; that is, planar multipolar cells (T stellate cells) of the PVCN receive auditory afferent input from the noise-stimulated ear and provide excitatory input to MOC neurons located in the superior olivary complex [77]; (2) increased responsiveness of MOC neurons, as might be mediated by the large, presumably, excitatory endings onto the cells that may represent descending inputs from the auditory cortex [78]; (3) increased efficacy of any or all of the synapses in the chain of MOC feedback to the tested cochlea, including between MOC terminals and outer hair cells. However, an explanatory mechanism for the increased MOCR strength remains a matter of further investigation.

Animal studies might be relevant to the discussion of NEB modifying the activity of the MOCR circuit. Research has shown that animals exposed to intense noise developed increased spontaneous activity in the ventral cochlear nucleus neurons unit types, which project to MOC neurons of the medial superior olivary complex [79]. The elevated activity that develops in onset choppers and transient choppers has been suggested to be associated with the increased activation of the MOCR circuit because both of these unit types are part of the MOCR circuit [80–82]. The animal data suggest that hyperactivity can develop within the MOCR circuit and may modify the strength of the MOCR after acoustic trauma.

### Functional significance of increased MOCR strength

The function of the increased MOCR strength remains a matter of further investigation. The MOCR strength has been positively correlated with susceptibility to permanent threshold shift (PTS) and temporary threshold shift (TTS) [83–85]. Several animal studies have shown that the MOCR strength can protect cochlear structures from noise-induced damage [86–88]. Electrical or acoustic stimulation to MOC fibers has been demonstrated to decrease the amount of PTS after noise exposure [86, 87], while sectioning of the MOC bundle increases the amount of PTS [88, 89]. These findings have been replicated in a human study finding significant negative correlation between strength of the MOCR and TTS induced by white noise [90]. At the same time, a few studies have failed to identify a relationship between the MOCR and susceptibility to TTS and PTS [91–93]. A recent field study which controlled important methodological variables found significant correlation between the strength of the MOCR and TTS in violinists [83]. In summary, there is evidence in the literature indicating a protective effect of the MOCR circuit on noise-induced cochlear damage. Therefore, it is likely that increased MOCR strength might contribute to protect cochlea from acoustic injury.

The role of conditioning exposures as a modulator of NIHL susceptibility is described well in the literature [94]. Noise conditioning can elevate sound evoked discharge rate of the MOC neurons [95]. This evidence suggests that the MOC neurons show long-term plasticity in acoustic responsiveness that is dependent on NEB [95]. Therefore, it can be hypothesized that increased strength of the MOCR in subjects with high NEB might be a temporary “top-down” adjustment to protect the cochlear mechanism from noise-induced damage. In other words, the conditioning sound might increase the MOCR strength to exert an otoprotective effect.

It is possible that the MOCR system might achieve normal functioning if acoustic overexposure is eliminated or reduced especially because there is evidence that cochlear injuries can induce temporary excitotoxicity in the auditory brainstem and midbrain [96]. The temporary excitotoxicity in the auditory brainstem is likely to be mediated by the MOC system [96]. Contrary to the temporary excitotoxicity, increased MOCR strength in patients with tinnitus and SLT might be permanent and pathological [37, 38]. The temporary excitotoxicity in the MOCR circuit might become permanent if noise exposure is not removed or reduced. Therefore, increased strength of the MOCR might be considered a risk factor to tinnitus and hyperacusis. Further research is needed to test these hypotheses.

### Experimental caveats

This study investigating the relationship between NEB and MOCR strength was limited by its survey design to estimate NEB. Although NEB was estimated using a validated survey tool, measurements using a comprehensive battery of noise dosimetry would yield greater precision. The questionnaire did not include an exhaustive list of noise exposure areas and it did not account for the use of ear protection in the process of calculating NEB score. The present study utilized a non-invasive CEOAE-based assay to measure the MOCR strength. This method allows measurement of fast MOC effects while slow MOC effects remain largely unassessed [97, 98].

## Conclusions

High NEB is associated with the increased MOCR strength in subjects with normal hearing sensitivity. It was hypothesized that (1) the increased MOCR strength is a temporary “top-down” adjustment to protect cochlea from noise damage; and (2) the temporary excitotoxicity in the MOCR circuit might become permanent if noise exposure is not removed or reduced. In this case, increased MOCR strength might be considered as a clinical risk factor for tinnitus and SLT. Further research is needed to investigate the functional significance of the relationship between NEB and the MOCR strength.

## Supporting information

S1 Appendix Questionnaires. (PDF)

S1 File Raw data. (SAV)
