# Supplementary material for: Increased medial olivocochlear reflex strength in normal-hearing, noise-exposed humans
Source: PLoS One. 2017 Sep 8;12(9):e0184036. doi: 10.1371/journal.pone.0184036 (PMC5590870; doi:10.1371/journal.pone.0184036)
Supplement: S1 Appendix — (PDF) [file pone.0184036.s001.pdf]

S1 Appendix: Questionnaires

Opening Questions:

1. Date: ______________

2. What is your age in years? ___________

3. What is your gender?
☐Male
☐Female
☐No disclosure

4. What is your ethnicity? Please check all that apply.

☐Black/ African American/ Afro Caribbean	☐Middle Eastern
☐White	☐South Asian
☐Hispanic/ Latino	☐East Asian
☐Native Hawaiian/Other Pacific Islander	☐Other ______________
☐American Indian/ Alaskan Native	☐I wish to not disclose

5. List any medications you take on a regular basis, including aspirin or herbal supplements.
☐I do not take any medications on a daily basis.
☐I take the following medications on a daily basis:

____

____

6. Do you have any allergies? ☐Yes ☐No

If yes, please list your allergies:

____

7. What illnesses do you have or have you had? Please check all that apply.

☐Meningitis	☐Heart Trouble
☐High Blood Pressure	☐Malaria
☐Head Injury	☐Scarlet Fever
☐Diabetes	☐None of the Above
☐Mumps	☐Other ______________

8. Do you have hearing loss?
☐Yes ☐No

If you answered “Yes” to question 8 please answer questions numbers 9- 12. If you answered “No” skip to question 13.

9. In which ear do you have problems with your hearing?
☐Right ☐Left ☐Both

10. If you have problems with your hearing, at what age did you first notice hearing problems?
____

11. When your hearing loss first started was it:
☐Gradual ☐Sudden ☐Fluctuating

12. How do you define the nature of your hearing problem?
☐Improved ☐Worsened ☐Stayed the same

13. Do you have a history of ear infections?
☐Yes ☐No ☐Don't know

14. Ever had 3 or more ear infections?
☐Yes ☐No ☐Don't know

15. If yes, did you receive successful treatment for your ear injury/ infection?
☐Yes ☐No

16. Have you ever had ear surgery?
☐Yes ☐No

17. If yes, in which ear have you had surgery?
☐Right ☐Left ☐Both

18. Have you ever had pressure equalization tubes?
☐Yes ☐No

19. If yes, in which ear have you had pressure equalization tubes?
☐Right ☐Left ☐Both

20. What is/was your occupation? If retired or other, please include your occupation. You may select more than one occupation if needed.

☐Health Professional
☐Engineer
☐Factory Worker
☐Musician
☐Administrative
☐Educator
☐Business Owner
☐Firefighter
☐Military Veteran
☐Retired
☐Other ____________
☐N/A
☐Student

21. What is your eye color?
☐Blue ☐Green ☐Gray ☐Hazel ☐Brown ☐Black

Noise/Music Exposure

Please answer these general questions about your hearing and any loud sounds. **DURING THE PAST YEAR (12 MONTHS):**

22. How often were you around or did you shoot firearms such as rifles, pistols, shotguns, etc.?

☐ Never ☐ Every Few Months ☐Monthly ☐ Weekly ☐ Daily

23. How often were you exposed to loud sounds while working on a paid job? By loud sounds, we mean sounds so loud that you had to shout or speak in a raised voice to be heard at arm's length.

☐ Never ☐ Every Few Months ☐Monthly ☐ Weekly ☐ Daily

24. How often were you exposed to any other types of loud sounds, such as power tools, lawn equipment, or loud music? By loud sounds, we mean sounds so loud that you had to shout or speak in a raised voice to be heard at arm's length.

☐ Never ☐ Every Few Months ☐Monthly ☐ Weekly ☐ Daily

25. How often were you exposed to loud sounds that made your ears "ring" or "buzz"?

☐ Never ☐ Every Few Months ☐Monthly ☐ Weekly ☐ Daily

26. How often were you exposed to loud sounds that made your hearing seem muffled for a while?

☐ Never ☐ Every Few Months ☐Monthly ☐ Weekly ☐ Daily

27. How often were you exposed to louds sounds that made your ears hurt, feel "full", or bother you in any other way?

☐ Never ☐ Every Few Months ☐Monthly ☐ Weekly ☐ Daily

28. Outside of a paid job, how often did you use power tools, chainsaws, or other shop tools?

☐ Never ☐ Every Few Months ☐Monthly ☐ Weekly ☐ Daily

If you used power tools, on average, how many hours did each time/ session last?

☐ 8 hours or more ☐4 hours up to 8 hours ☐ 1 hour up to 4 hours ☐ Less than 1 hour

If you used power tools, how often did you wear earplugs or earmuffs during this activity?

☐ Never ☐ Sometimes ☐ Always

29. Outside of a paid job, how often did you drive heavy equipment or use loud machinery (such as tractors, trucks, or farming or lawn equipment like mowers/ leaf blowers)?

☐ Never ☐ Every Few Months ☐Monthly ☐ Weekly ☐ Daily

If you drove/ used loud machinery, on average, how many hours did each time/ session last?

☐ 8 hours or more ☐4 hours up to 8 hours ☐ 1 hour up to 4 hours ☐ Less than 1 hour

If you drove/ used machinery, how often did you wear earplugs or earmuffs during this activity?

☐ Never ☐ Sometimes ☐ Always

30. How often did you attend car/ truck races, commercial/ high school sporting events, music concerts/ dances, or any other events with amplified public announcement (PA)/ music systems?

☐ Never ☐ Every Few Months ☐Monthly ☐ Weekly ☐ Daily

If you attended these events, on average, how many hours did each time/ session last?

☐ 8 hours or more ☐4 hours up to 8 hours ☐ 1 hour up to 4 hours ☐ Less than 1 hour

If you attended these events, how often did you wear earplugs or earmuffs during this activity?

☐ Never ☐ Sometimes ☐ Always

31. How often did you ride/ operate motorized vehicles such as motorcycles, jet skis, speed boats, snowmobiles, or four wheelers?

☐ Never ☐ Every Few Months ☐Monthly ☐ Weekly ☐ Daily

If you rode motorized vehicles, on average, how many hours did each time/ session last?

☐ 8 hours or more ☐4 hours up to 8 hours ☐ 1 hour up to 4 hours ☐ Less than 1 hour

If you rode motorized vehicles, how often did you wear earplugs or earmuffs during this activity?

☐ Never ☐ Sometimes ☐ Always

32. How often did you ride in or pilot small aircrafts/ private airplanes?

☐ Never ☐ Every Few Months ☐Monthly ☐ Weekly ☐ Daily

If you flew airplanes, how many hours did each time/ session last?

☐ 8 hours or more ☐4 hours up to 8 hours ☐ 1 hour up to 4 hours ☐ Less than 1 hour

If you flew airplanes, how often did you wear earplugs or earmuffs during this activity?

☐ Never ☐ Sometimes ☐ Always

33. How often were you around or did you shoot firearms such as rifles, pistols, shotguns, etc.

☐ Never ☐ Every Few Months ☐Monthly ☐ Weekly ☐ Daily

If you were around/ shot firearms, on average, how many shots did you fire each time/ session?

___________ Shotgun/ rifle shots per session ___________ pistol shots per session

If you were around/ shot firearms, how often did you wear earplugs or earmuffs while shooting?

☐ Never ☐ Sometimes ☐ Always

34. How often were you around firecrackers or other fireworks?

☐ Never ☐ Every Few Months ☐Monthly ☐ Weekly ☐ Daily

If you were around fireworks, on average, how many fireworks did you shoot each time/ session?

___________ Firecracker/ firework shots per session

If you were around/ shot fireworks, how often did you wear earplugs or earmuffs during this activity?

☐ Never ☐ Sometimes ☐ Always

35. How often did you play a musical instrument?

☐ Never ☐ Every Few Months ☐Monthly ☐ Weekly ☐ Daily

If you played, please tell us what musical instrument:

____

If you played a musical instrument, on average, how many hours did each time/ session last?

☐ 8 hours or more ☐4 hours up to 8 hours ☐ 1 hour up to 4 hours ☐ Less than 1 hour

If you played a musical instrument, how often did you wear earplugs or earmuffs while playing?

☐ Never ☐ Sometimes ☐ Always

36. How often did you listen to music, radio programs, etc. using personal <u>headsets</u> or <u>earphones</u>?

☐ Never ☐ Every Few Months ☐Monthly ☐ Weekly ☐ Daily

If you listened through earphones, on average, how many hours did each time/ session last?

☐ 8 hours or more ☐4 hours up to 8 hours ☐ 1 hour up to 4 hours ☐ Less than 1 hour

If you listened through earphones, what was the typical volume setting (control knob rotation) when listening?

☐ Full/ maximum volume ☐ ¾ maximum volume ☐ ½ max. volume ☐ ¼ max. volume

37. Other than music concerts and headset use (*already covered in questions 9 and 15),* how often did you listen to music, radio programs, etc. rom audio speakers in a car or at home?
☐ Never ☐ Every Few Months ☐Monthly ☐ Weekly ☐ Daily

If you listened via speakers, on average, how many hours did each time/ session last?

☐ 8 hours or more ☐4 hours up to 8 hours ☐ 1 hour up to 4 hours ☐ Less than 1 hour

If you listened via speakers, what was the typical volume setting (control know rotation)?

☐ Full/ maximum volume ☐ ¾ maximum volume ☐ ½ max. volume ☐ ¼ max. volume

38. Now think back to this past summer. Over the summer months, did you work a noisy paid job, such as construction, farming, a factory, lawn service, carwash, or other indoor or outdoor job working around loud equipment or machinery? By noisy job, we mean sounds so loud that you had to shout or speak in a raised voice to be heard at arm's length.

☐ Yes ☐ No (If no, skip to question #39)

If yes, please describe this noisy job:
____

If you worked a noisy job, please estimate the number of hours you worked in a typical week:

________________________ hours worked per typical week this summer

If you worked a noisy job this summer, did your employer give you earplugs or earmuffs to wear at work?

☐ Yes ☐ No

How often did you wear earplugs or earmuffs when around loud noises at this summer job?

☐ Never ☐ Sometimes ☐ Always

Did you receive training on this job about noise and hearing loss? ☐ Yes ☐ No

Did you receive a hearing test through this job? ☐ Yes ☐ No
Other comments:

39. Other than during the summer, over the past year, did you work one or more noisy paid jobs, such as construction, farming, a factory, lawn service, carwash, or other indoor or outdoor job working around loud equipment or machinery? By noisy job, we mean sounds so loud that you had to shout or speak in raised voice to be heard at arm's length.

☐ Yes ☐ No

If yes, please describe the noisy jobs: ____

If you worked a noisy job, please estimate the number of hours you worked in a typical week:

_______________________ average hours worked per typical week during the school year

If you worked a noisy job during the school year, did your employer give you earplugs or earmuffs to wear at work?

☐ Yes ☐ No

How often did you wear earplugs or earmuffs when around loud noises at this noisy job(s)?

☐ Never ☐ Sometimes ☐ Always

Did you receive training on this job about noise and hearing loss? ☐ Yes ☐ No

Did you receive a hearing test through work? ☐ Yes ☐ No
Other comments:

Smoking

40. Do you or have you smoke tobacco? (If no, skip to question #44)
☐Yes ☐No

41. What types of smoking do you prefer, or have preferred, on a regular basis? (percentage values of all selected choices must add up to 100%)

☐Cigarettes ☐Cigars or spliffs
☐Cigarillos ☐Pipe
☐Tobacco ☐Water pipe
☐Other (please specify) ______________________________

42. Please list the cigarettes/cigars/marijuana brands you prefer, or have preferred, on a regular basis for

Preferences	Brand name
Preference #1	
Preference #2	
Preference #3	

43. How many number of below items you would say you smoked in a typical day, and how many years you smoked?

Type	Number per day	Number of years
Preference #1		
Preference #2		
Preference #3		

Rate Your Agreement

44. Many everyday sound are unbearably loud to me (please rate your agreement with this statement on 0 to 100 scale, 0 = completely disagree and 100 = completely agree)

____

45. Sounds that others believe are moderately loud are too loud for me(please rate your agreement with this statement on 0 to 100 scale, 0 = completely disagree and 100 = completely agree)

____

46. I hear very soft sounds that others with normal hearing do not hear (please rate your agreement with this statement on 0 to 100 scale, 0 = completely disagree and 100 = completely agree)

____

Tinnitus

47. In the **past 12 months**, have you been bothered by ringing, roaring, or buzzing in ears or head **that lasts for 5 minutes or more**?

☐Yes ☐No

48. If you answered "Yes" to Q#47, then how long have you been bothered by this ringing, roaring, or buzzing in ears or head?

☐Less than three months ☐Three months to a year
☐1 to 4 years ☐5 to 9 years
☐10 or more years ☐Don't know

49. Have you ever experienced ringing, roaring, or buzzing in your ears/head?
☐Yes ☐No

If your answer is "No" to question #49, please skip the remaining survey

50. Are you bothered by ringing, roaring, or buzzing in your ears or head only after listening to loud sounds or loud music?

☐Yes ☐No ☐Don't know

51. How much of a problem is this ringing, roaring, or buzzing in your ears or head?

☐ No problem ☐ A small problem
☐ A moderate problem ☐ A big problem
☐ A very big problem ☐ Don’t know

52. In which ear do you hear tinnitus (i.e. perception of sound in the absence of external sound)?
☐ Right ☐ Left ☐ Both ears or head

53. What best describes the sound that you hear? Please check all that apply.
☐ Pulsating ☐ Buzzing
☐ Roaring ☐ Rushing water
☐ Ringing ☐ Crickets
☐ Hissing ☐ Other ____________

54. On a scale of 0-100, how loud is the sound you hear? (0= very soft, 100= painfully loud)

____

55. How do you describe your tinnitus perception?
☐ Continuous
☐ Intermittent
☐ Other ________________________

56. How did your tinnitus start?

☐ After noise exposure ☐ Not sure how it started
☐ After sleep ☐ After medication
☐ After music exposure ☐ Other ___________________

Tinnitus Handicap Inventory

Instructions: The purpose of this questionnaire is to identify, quantify, and evaluate tinnitus-related distress in daily living that you may be experiencing because of tinnitus. Please do not skip any questions.

#Q	Text	Check		
1	Because of your tinnitus, is it difficult for you to concentrate?	Yes	Sometimes	No
2	Does the loudness of your tinnitus make it difficult for you to hear people?	Yes	Sometimes	No
3	Does your tinnitus make you angry?	Yes	Sometimes	No
4	Does your tinnitus make you feel confused?	Yes	Sometimes	No
5	Because of your tinnitus, do you feel desperate?	Yes	Sometimes	No
6	Do you complain a great deal about your tinnitus?	Yes	Sometimes	No
7	Because of your tinnitus, do you have trouble falling to sleep at night?	Yes	Sometimes	No
8	Do you feel as though you cannot escape your tinnitus?	Yes	Sometimes	No
9	Does your tinnitus interfere with your ability to enjoy your social activities (such as going out to dinner, to the movies)?	Yes	Sometimes	No
10	Because of your tinnitus, do you feel frustrated?	Yes	Sometimes	No
11	Because of your tinnitus, do you feel that you have a terrible disease?	Yes	Sometimes	No
12	Does your tinnitus make it difficult for you to enjoy life?	Yes	Sometimes	No
13	Does your tinnitus interfere with your job or household responsibilities?	Yes	Sometimes	No
14	Because of your tinnitus, do you find that you are often irritable?	Yes	Sometimes	No
15	Because of your tinnitus, is it difficult for you to read?	Yes	Sometimes	No
16	Does your tinnitus make you upset?	Yes	Sometimes	No
17	Do you feel that your tinnitus problem has placed stress on your relationships with members of your family and friends?	Yes	Sometimes	No
18	Do you find it difficult to focus your attention away from your tinnitus and on other things?	Yes	Sometimes	No
19	Do you feel that you have no control over your tinnitus?	Yes	Sometimes	No
20	Because of your tinnitus, do you often feel tired?	Yes	Sometimes	No
21	Because of your tinnitus, do you feel depressed?	Yes	Sometimes	No
22	Does your tinnitus make you feel anxious?	Yes	Sometimes	No
23	Do you feel that you can no longer cope with your tinnitus?	Yes	Sometimes	No
24	Does your tinnitus get worse when you are under stress?	Yes	Sometimes	No
25	Does your tinnitus make you feel insecure?	Yes	Sometimes	No
